# Tracking nutrients in space and time: Interactions between grazing lawns and drought drive abundances of tallgrass prairie grasshoppers

**DOI:** 10.1002/ece3.7435

**Published:** 2021-03-18

**Authors:** Katerina A. Ozment, Ellen A. R. Welti, Monica Shaffer, Michael Kaspari

**Affiliations:** ^1^ Geographical Ecology Group Department of Biology University of Oklahoma Norman OK USA; ^2^ Senckenberg Research Institute and Natural History Museum Frankfurt Gelnhausen Germany; ^3^ Kansas Department of Agriculture Manhattan KS USA

**Keywords:** Acrididae, bison, grassland, grazers, herbivore, Konza Prairie, nitrogen

## Abstract

1. We contrast the response of arthropod abundance and composition to bison grazing lawns during a drought and non‐drought year, with an emphasis on acridid grasshoppers, an important grassland herbivore.
2. Grazing lawns are grassland areas where regular grazing by mammalian herbivores creates patches of short‐statured, high nutrient vegetation. Grazing lawns are predictable microsites that modify microclimate, plant structure, community composition, and nutrient availability, with likely repercussions for arthropod communities.
3. One year of our study occurred during an extreme drought. Drought mimics some of the effects of mammalian grazers: decreasing above‐ground plant biomass while increasing plant foliar percentage nitrogen.
4. We sampled arthropods and nutrient availability on and nearby (“off”) 10 bison‐grazed grazing lawns in a tallgrass prairie in NE Kansas. Total grasshopper abundance was higher on grazing lawns and the magnitude of this difference increased in the wetter year of 2019 compared to 2018, when drought led to high grass foliar nitrogen concentrations on and off grazing lawns. Mixed‐feeding grasshopper abundances were consistently higher on grazing lawns while grass‐feeder and forb‐feeder abundances were higher on lawns only in 2019, the wetter year. In contrast, the abundance of other arthropods (e.g., Hemiptera, Hymenoptera, and Araneae) did not differ on and off lawns, but increased overall in 2019, relative to the drought of 2018.
5. Understanding these local scale patterns of abundances and community composition improves predictability of arthropod responses to ongoing habitat change.

## Graphical abstract

Tallgrass prairie grasshoppers live surrounded by their food, but much of the available forage is nutrient poor. We report increases in grasshopper abundance and shifts in composition in response to both grazing lawns, high nutrient patches created by mammalian grazers, and drought, which concentrates nutrients in tallgrass prairie foliage.

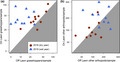

## 1 INTRODUCTION

Grazing and climate, along with fire, are major drivers shaping grassland communities and ecosystems. Large mammalian herbivores can reshape grassland communities by consuming plants and redistributing nutrients. Grazing reduces aboveground plant biomass, leads to morphological differences in plant structure, restructures plant community composition, increases soil compaction, and alters nutrient availability, all of which may, in turn, affect arthropod communities (Joern, 2005; van Klink et al., 2013; van der Plas & Olff, 2014). Some mammals create **grazing lawns**: areas regularly grazed by large herbivores creating patches of short‐grass swards which can act as nutrient hotspots (Hempson et al., 2015; Mayengo et al., 2020; McNaughton, 1984). Grazing lawns are often maintained for years to decades by a positive feedback loop, in which high‐quality forage encourages further grazer visits (McNaughton, 1984; Veldhuis et al., 2016), and grazer visits enhance limiting nutrients like nitrogen, potassium, and sodium in grazing lawn plants either through their excretions (Steinauer & Collins, 1995) or altering plant‐soil water balance (Veldhuis et al., 2014). The low plant stature, and increased solar insolation, generates unique, dependable habitat patches (van Klink et al., 2015) with potential effects on insect community abundance and composition (Purdon et al., 2019).

**Periodic drought** is another feature of grasslands that can generate microclimate and nutrient effects similar to grazing lawns. Drought can concentrate nutrient levels in foliage (e.g. Aaltonen et al., 2017; Augustine et al., 2018) both as a result of changes in community composition favoring plant species with increased nutrient use efficiency (Luo et al., 2018) or via lower plant production resulting in total nutrients concentrated in less biomass (Grant et al., 2014). Watering experiments suggest plant foliar percentage nitrogen increases in drought conditions, and grasshoppers‐fed drought‐stressed plants grow faster and larger (Franzke & Reinhold, 2011). Moreover, in the Plant Stress Hypothesis (PSH, White, 1969), herbivore performance is enhanced when stressors like drought increase foliar nitrogen due to either altered nutrient transport (Behmer & Joern, 2012; Joern & Mole, 2005) or changes in secondary metabolite content of plant species or plant parts (Cornelissen et al., 2008; Gutbrodt et al., 2011; Huberty & Denno, 2004).

Both drought and grazing lawns are thus likely to shape the abundance and composition of short‐horned grasshoppers (Acrididae), a dominant grassland herbivore that can consume aboveground plant biomass in amounts comparable to cattle at USDA stocking rates (Onsager, 2000). Here, we use a 2‐year study to exploit two “natural experiments”—bison grazing lawns and an extreme drought year followed by year of average rainfall—to address how drought interacts with large mammalian grazing to determine local grasshopper abundances at this same protected tallgrass prairie in northeastern Kansas. Previous work in this system has shown that grasshopper diversity and abundance increases in areas grazed by bison compared to ungrazed sites (Joern, 2005; Welti, Qiu, et al., 2019), but the local effects of grazing lawns on the grasshopper community have yet to be investigated. Additionally, an analysis of long‐term grasshopper abundances at KPBS revealed that drought can increase grasshopper abundances (Welti et al., 2020). We test the hypothesis that high nutrient quality of forage—driven by grazing lawns, drought, or both—attract higher abundances of arthropods, especially the herbivorous and highly mobile grasshoppers. We thus contrast two drivers of nutritional heterogeneity on grassland arthropods: grazing lawns, which create high plant nutrient patches in space, and drought, which can increase plant nutrient concentrations in time.

## 2 MATERIALS AND METHODS

### 2.1 Site description

Konza Prairie Biological Station (KPBS) is a 3,487‐hectare native tallgrass prairie and Long Term Ecological Research (LTER) site in NE Kansas, USA. KPBS has high grasshopper species diversity with over 40 species recorded and around 20 species sampled with regularity (Joern, 2018). Bison (*Bison bison*) were reintroduced to KPBS in 1987, leading to the redevelopment of grazing lawns. Bison currently occupies a 949‐ha area of KPBS and the herd is maintained at 260 individuals fluctuating with ~90 calves born each spring and a fall culling (Raynor et al., 2016). Since 1972, KPBS has had a system of controlled burns, with watersheds subjected to mostly spring burning at different historical fire intervals (i.e. burned every 1, 2, 4, and 20 years).

KPBS has a temperate continental climate, with an average annual cumulative precipitation of 835 mm, predominately falling in spring. Local climate data collected at the KPBS meteorological station are archived by the Climate and Hydrology Database project (https://climhy.lternet.edu) with support from NSF Long Term Ecological Research (LTER) and the USDA Forest Service. KPBS experienced a severe drought during the growing season months (March–July) of 2018 with only 263 mm of cumulative precipitation. This is only 55% of the long‐term (1983–2019) average of 477 mm of growing season precipitation and the driest KNZ growing season since 1989 (212 mm). Comparatively, KPBS had 544 mm (114% of long‐term average), 405 mm (85% of long‐term average), and 647 mm (136% of long‐term average) of precipitation during the growing seasons of 2016, 2017, and 2019, respectively. Monthly cumulative precipitation and mean monthly temperatures from January 2017 to July 2019 (month of final arthropod sampling) are shown in Fig. S1.

### 2.2 Grazing lawns

Grazing lawns were identified visually in the springs of 2016–2018, totaling 13 grazing lawns from across the KPBS bison area (Shaffer, 2019). Grazing lawns are highly distinct from surrounding grassland, especially in spring, and at KPBS tend to occur either on hilltops or hillsides (Fig. S2). In 2018, we selected the 10 most active grazing lawns for arthropod sampling, with lawns occurring in watersheds subjected to fire return intervals of every 2, 4, and 20 years (Table S1).

### 2.3 Arthropod sampling

Grasshoppers and other arthropods were collected on each grazing lawn (“on lawn” sites), and a paired location that was selected haphazardly, ~100 m from the grazing lawn, but importantly, still within the bison area (“off lawn” sites). Arthropod samples, consisting of 40 sweeps of a 38 cm diameter sweepnet, were collected twice on each grazing lawn and twice on each paired off grazing lawn area. Sampling was conducted on warm, still, and sunny afternoons in early July of 2018 and 2019. Once collected, samples were placed in a cooler in the field, then freeze‐killed in the lab. Whereas in early spring, grazing lawn vegetation was heavily grazed and too short‐statured to efficiently use sweepnet sampling; by July, bison had more widespread grazing preferences and grazing lawns had sufficient vegetation to conduct sweepnet sampling. While sweepnets characterize relative abundance rather than true density (Joern, 2004), our consistent sampling methods allow us to compare grasshopper abundances on paired on lawn and off lawn sites and between years. We identified grasshoppers to species. Non‐grasshopper arthropods were categorized into eight taxonomic/functional groups consisting of herbivorous Hemiptera, predatory Hemiptera, pollinators, Tettigoniidae, Formicidae, parasitoid Hymenoptera, Araneae, and all other arthropods.

### 2.4 Soil and plant samples

Plant and soil chemistry on KPBS grazing lawns and paired off lawn locations were collected for a previous unpublished study and are analyzed here to evaluate grazing lawn effects on nutrient availability. These data are used with two caveats: (a) Plant and soil data were not collected simultaneously with arthropod sampling. Soils were collected for nutrient analysis in 2017, and plants were collected for biomass and percentage nitrogen in 2016–2018. (b) Grazing lawns sampled for plants varied each year (2016 lawns: 1, 2, 3, 4, and 5; 2017 lawns: 5, 6, 7, 8, 9; 2018 lawns: 1, 6, 10, 11, 12; see Table S1). Grazing lawns were selected each year based on those that were most heavily used by bison in the spring. Thus, plant and soil data cannot be directly compared with arthropod sampling. However, these data provide valuable background information on the effects of grazing lawns on soils and plants.

Soil samples were collected using a T‐shape corer with a depth of 30 cm from five grazing lawns (lawns 5, 6, 7, 8, and 9) in late June and early July. For each grazing lawn, five subsamples of soil were collected, each with a dry weight ranging from 22 to 67 g. Grass was clipped from six 0.9 m^2^ quadrats on five grazing lawns and corresponding paired off lawn locations from 2016 to 2018. Plant samples were dried at 60°C for a minimum of 48 hr, and grasses and forbs were then each weighed to obtain dry mass. Grass samples were analyzed for total percentage nitrogen by combustion analysis. Soil samples were analyzed for concentrations of phosphorous using the Mehlich 3 extractant, sodium determined by the ammonium acetate method, and ammonium and nitrate using KCl extraction performed on a Rapid Flow Analyzer (Model RFA‐300). Plant and soil chemical analysis was conducted at the Kansas State University Soil Testing Lab (https://www.agronomy.k‐state.edu/services/soiltesting/).

### 2.5 Arthropod abundance analysis

For all arthropod abundance analyses, the two subsamples collected at each location (either on or off each grazing lawn) each year were summed to total abundance sampled/location/year to reduce pseudoreplication and log‐transformed to reduce heteroscedasticity. In order to examine the effects of grazing lawn and year on grasshopper abundance, we ran linear mixed models with predictor variables including the fixed effects of grazing lawn treatment (on or off lawn), year, and an interaction of these terms. Initial analyses found no significant effects of the fire return interval (total grasshoppers: *F*_1,34_ = 0.3, *p* = .59) or years since last burn (total grasshoppers: *F*_1,34_ = 1.2, *p* = .28), and thus these terms were not included in the final models. This result is consistent with previous work on KPBS grasshopper which has shown fire frequency has strong effects on grasshoppers in the absence of grazing, but only weak effects in bison‐grazed grassland (Welti, Qiu, et al., 2019). We did not include transect in our model as abundances were summed within each location/year with location being our replicate level. Grazing lawn pair identity was included as a random effect to account for both our paired design and sampling the same lawns across time (Zuur et al., 2009). The grasshopper species abundance matrix was log + 1 transformed prior to analysis. Mixed model responses included abundances of total grasshoppers, within feeding guilds (forb‐feeder, grass‐feeder, and mixed‐feeder, following Jonas & Joern, 2007) and within subfamilies (Gomphocerinae, Melanoplinae, and Oedipodinae). To visualize mixed model results, we plotted sampled abundance on each grazing lawn for each year over the sampled abundance of the paired location off lawn.

We additionally examined responses of abundances of total non‐grasshopper arthropods and the more abundant (>100 sampled individuals) sorted taxa of herbivorous Hemiptera, Tettigoniidae, Formicidae, parasitoid Hymenoptera, and Araneae using mixed models with the same predictor variables used to examine grasshopper abundance responses.

### 2.6 Grasshopper composition analysis

We used a partial Redundancy Analysis (RDA) to examine responses of grasshopper community composition. Our full a priori model included the fixed predictor variables of grazing lawns (on or off), year, the historical burn frequency, and the number of years since the last time the watershed was burned. The RDA is considered partial as it is conditional on grazing lawn pair identity to account for the paired sampling design and repeated sampling of the same locations over time. We conducted forward stepwise selection to identify which fixed predictor variables significantly affected grasshopper community composition. RDA is a standard ordination technique in community ecology for testing the effects of habitat characteristics on community species composition (Legendre & Anderson, 1999). RDA analysis was conducted using the R package vegan (Oksanen et al., 2018).

### 2.7 Soil and plant analysis

To test for differences in soil chemistry on and off grazing lawns in 2017, we ran linear mixed models with a fixed effect of grazing lawn treatment (on or off lawn) and a random effect of grazing lawn pair identity. To test for differences in plant biomass and grass percentage nitrogen due to grazing lawns or climate conditions over years, we ran mixed models including fixed effects of grazing lawn treatment (on or off lawn) and year as a random effect of grazing lawn pair identity. To examine the relationship between grass biomass and grass percentage nitrogen, we ran a mixed model, using on/off grazing lawn site ID as the random variable. All mixed models were run using the R package lme4 (Bates et al., 2015).

All analyses were run in R version 3.6.1 (R Core Team, 2020).

## 3 RESULTS

### 3.1 Grasshopper abundance

**FIGURE 1:**
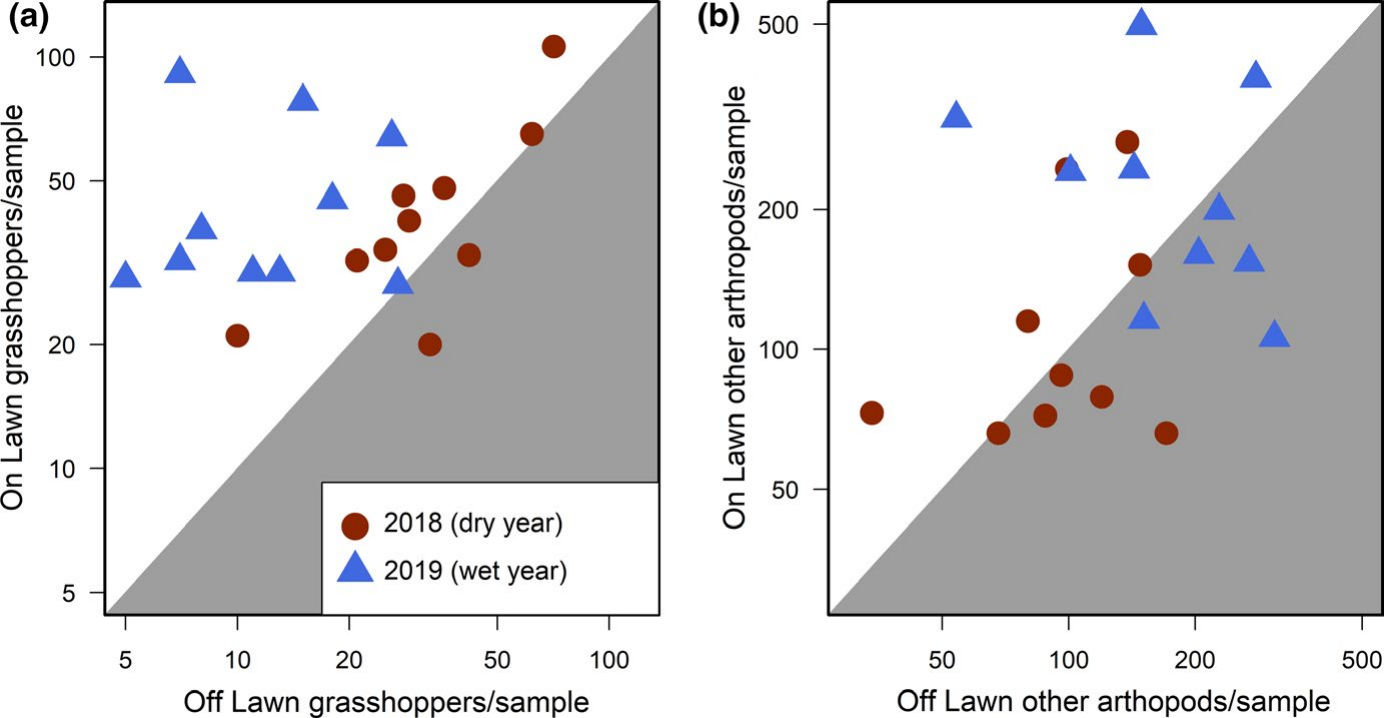
Grasshoppers were abundant everywhere in the dry year (2018); however, in the wetter year (2019), grasshoppers were only abundant on grazing lawns (a). Other arthropods did not response to grazing lawns but increased in the wetter year of 2019 (b). Each point represents a paired comparison of the average abundance per sample collected on a grazing lawn and within a sampling year over the average abundance per sample collected on the corresponding paired off lawn site. Model statistics for abundance responses to grazing lawn and year are provided in Table 1

We sampled 1,404 total grasshoppers from 21 species across all locations in both the drought year of 2018 and 2019 (Table S2). Grasshopper abundances varied as an interaction between grazing lawn and year (Figure 1a; Table 1A) and were higher in the drought year of 2018 while grazing lawns increased abundances more in the wetter year (2019 effect size = 1.9 versus 0.4 in 2018).

**TABLE 1:** Mixed model results for effects of grazing lawn (lawn) and year on total grasshopper abundance (A), and abundance of all other sampled arthropods (B), with *n* = 40 for both models

	Estimate	*SE*	*t*	*p*
(A) Grasshopper abundance				
Intercept	1.5	0.07	20.9	<.001
lawn	0.1	0.09	1.08	.28
year	0.42	0.09	4.64	<.001
lawn * year	0.45	0.13	3.51	<.001
(B) Other arthropod abundance				
Intercept	1.98	0.07	28.17	<.001
lawn	0.05	0.1	0.47	.64
year	−0.25	0.1	−2.48	.01
lawn * year	0.06	0.14	0.42	.68

Note

Table includes estimate, estimate standard error, *t*‐test statistic, and *p*‐value for each fixed variable and the intercept. Positive estimates for lawn indicate higher abundances on versus off grazing lawns. Positive estimates for year indicate higher abundances in 2018 (the drought year) compared to 2019. Abundance responses are visualized in Figure 1.

Within grasshopper feeding guilds, grassfeeding and forbfeeding grasshopper abundance were higher on grazing lawns only in the wetter year of 2019, whereas mixed‐feeding grasshopper abundance was higher on grazing lawns in both drought and normal drought (Table S3; Fig. S3). Within the subfamilies, Gomphocerinae had a higher abundance on grazing lawns in 2019, Melanoplinae did not respond to grazing lawns but were more abundant in 2018 sampling, and Oedipodinae, while less abundant than the other subfamilies, were consistently higher on grazing lawns (Table S3; Fig. S3).

### 3.2 Abundance of other arthropods

We sampled 6,577 non‐grasshopper arthropods over the two years of this study. Total non‐grasshopper arthropod abundance did not differ between grazing lawns and their paired controls, but increased overall in 2019 relative to the 2018 drought (Figure 1b; Table 1B). The same was true for common taxonomic groups of herbivorous Hemiptera, Tettigoniidae, Formicidae, and Araneae. Parasitoid Hymenoptera were more abundant in 2019 (Table S4).

### 3.3 Grasshopper composition

**FIGURE 2:**
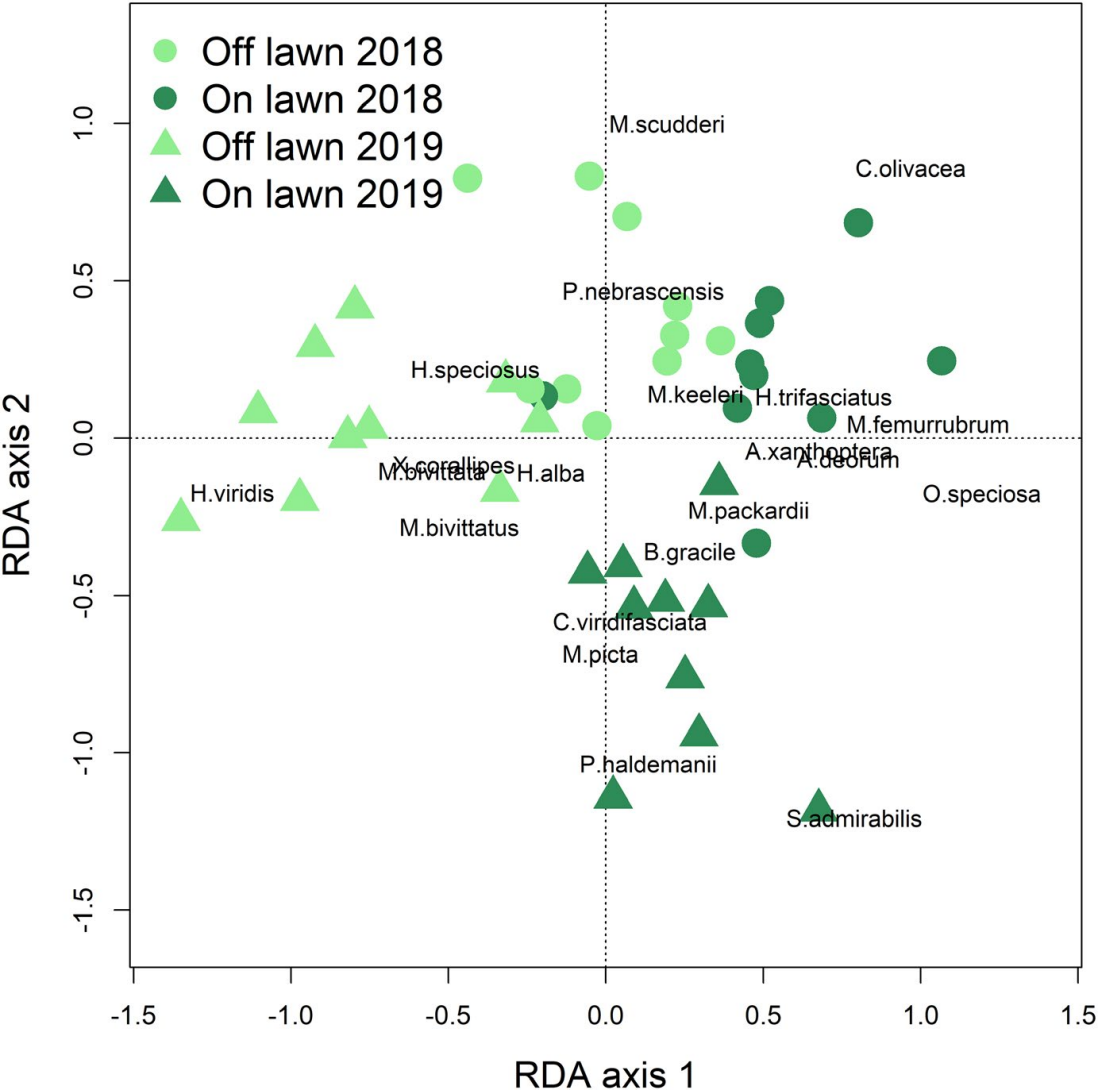
Biplot of the final partial Redundancy Analysis (RDA) model showing the response of grasshopper community composition to grazing lawns and year. Both the first (*F*_(1,28)_ = 3.72, Variance = 1.79, *p* = .001) and second (*F*_(1,28)_ = 1.76, Variance = 0.85, *p* = .039) RDA axes were significant; *F* distributions are based on 999 permutations. Corresponding ANOVA results for the partial RDA are included in Table S5

Grasshopper community composition differed on and off grazing lawns and between years (Figure 2; Table S5). Neither the historical burn frequency nor the number of years since the last burn affected grasshopper community composition and thus were removed from the partial RDA following stepwise selection. The final partial RDA model of grasshopper community composition, conditional on grazing lawn pair identity and including the fixed predictors of year and presence of grazing lawn accounted for variation in grasshopper community composition (*F*_(2,28)_ = 2.74, Variance = 2.64, *p* = .001; *F* distribution based on 999 permutations).

### 3.4 Toward mechanism: Verifying the effect of grazing lawns and drought on soil and plant nutrients

**FIGURE 3:**
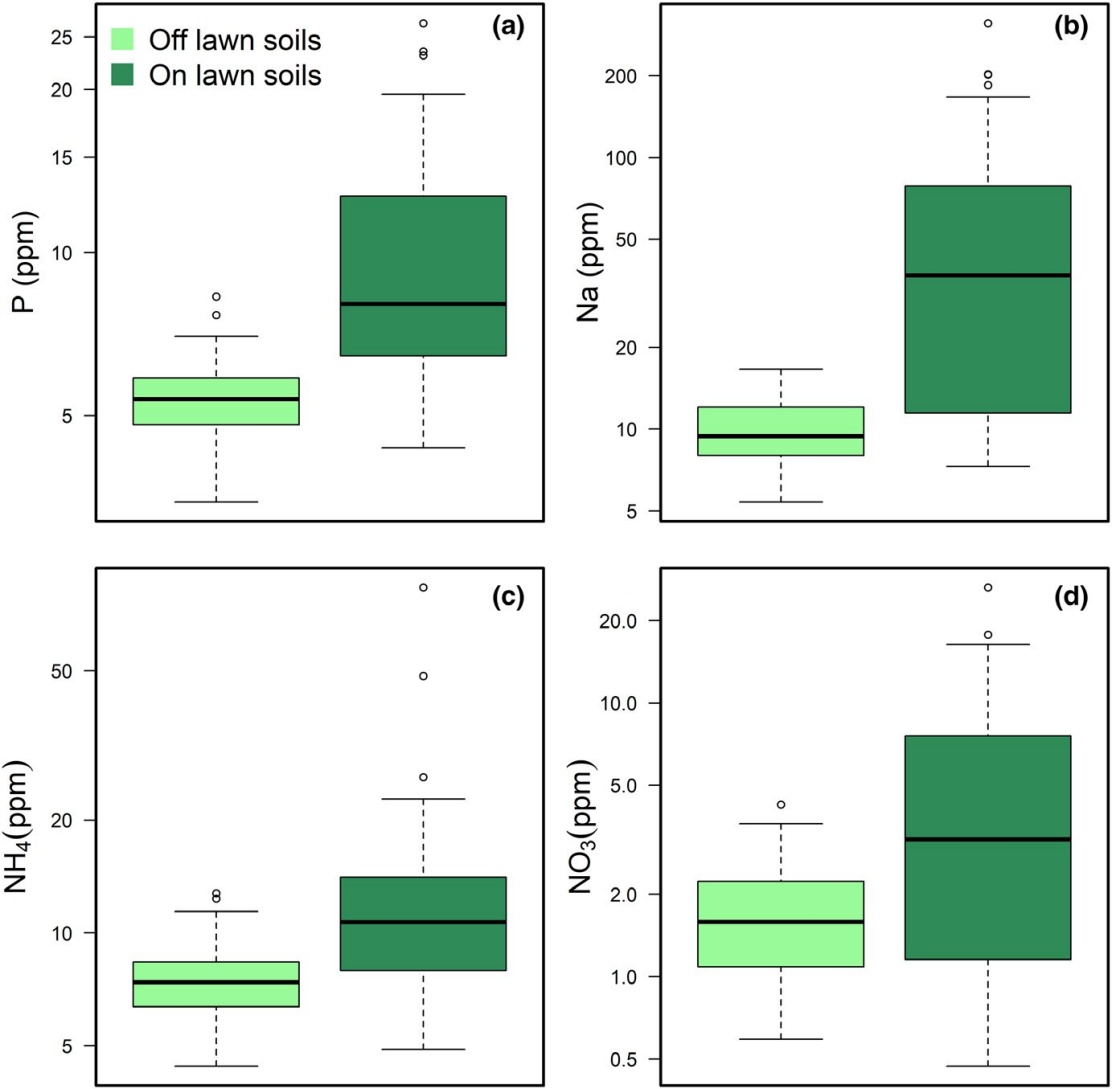
Soil chemistry from five KPBS grazing lawns from 2017 revealed grazing lawns had higher soil concentrations of phosphorus (a; Est. = 4.54, *SE* = 0.81, *t* = 5.59, *p* < .001), sodium (b; Est. = 52.56, *SE* = 10.83, *t* = 4.85, *p* < .001), ammonium (c; Est. = 6.65, *SE* = 2.08, *t* = 3.19, *p* = .001), and nitrate (d; Est. = 3.7, *SE* = 0.79, *t* = 4.72, *p* < .001) compared to nearby areas within the bison area but not on grazing lawns

**FIGURE 4:**
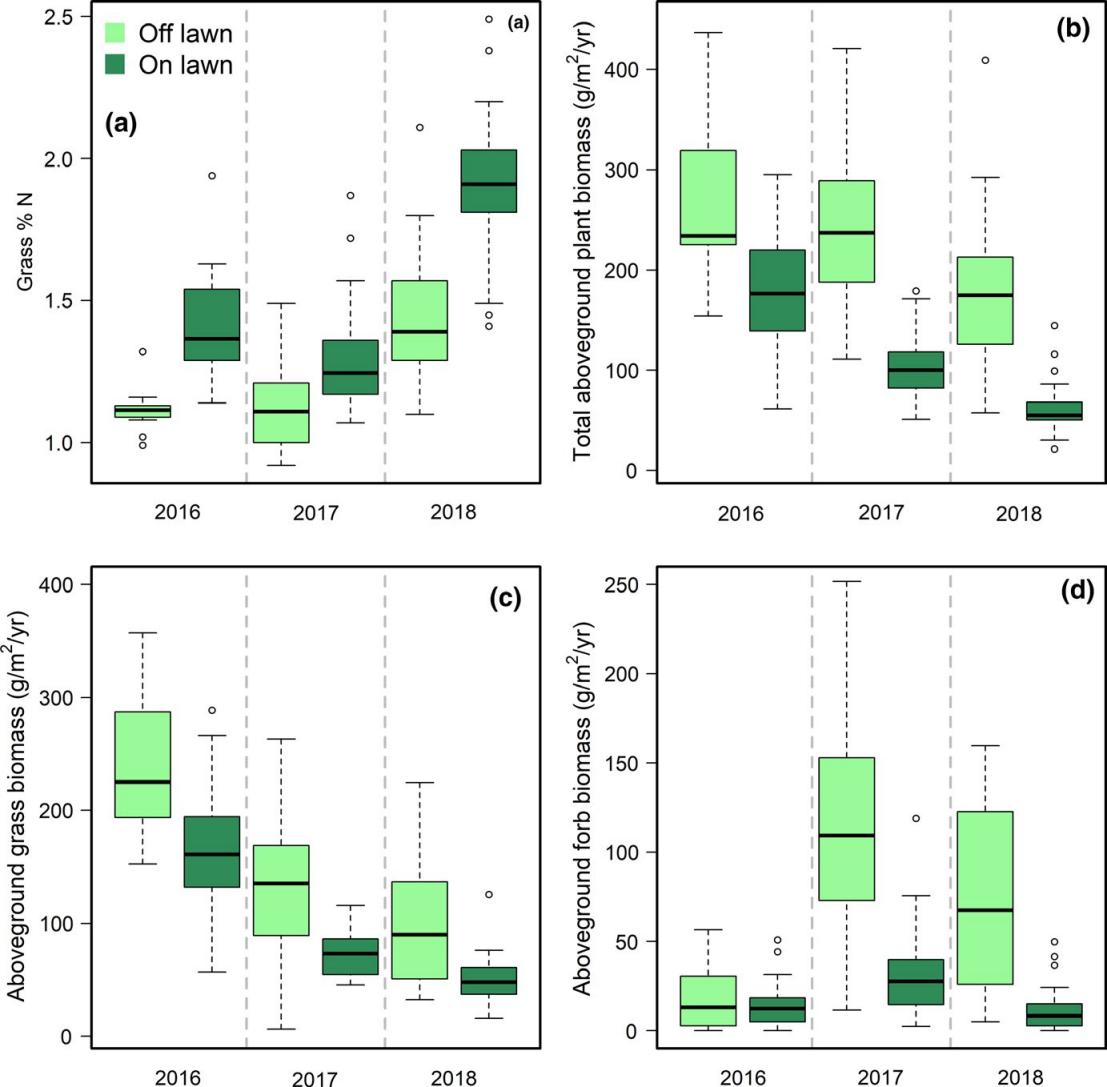
Grasses were analyzed for percentage nitrogen from five grazing lawns and five nearby locations in the bison area each year from 2016 to 2018 (a). Grass percentage nitrogen increased on grazing lawns and in the dry year of 2018, but was not significantly different between 2016 and 2017 (Table S6A). Total plant biomass (b), grass biomass (c), and forb biomass (d) were all significantly higher off grazing lawns than on grazing lawns and varied with sampling year (Table S6B–D)

**FIGURE 5:**
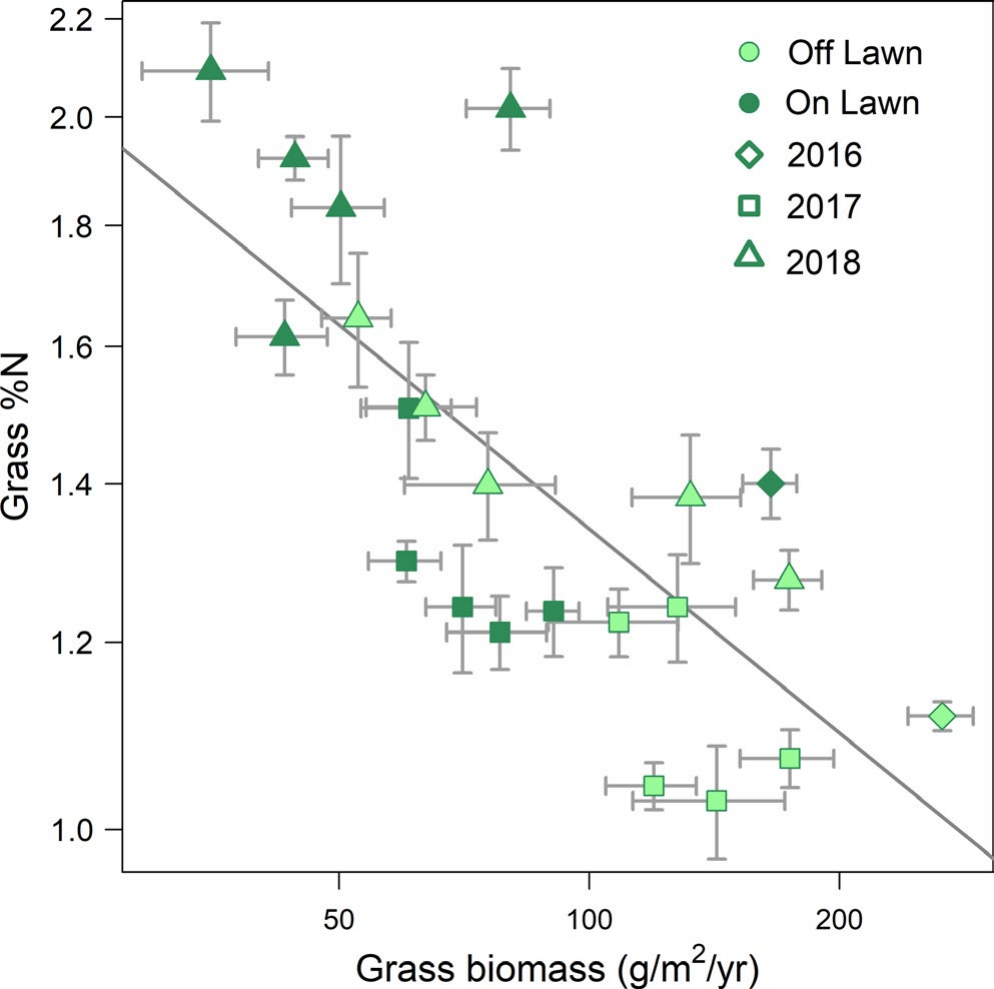
Grass percentage nitrogen decreased with grass biomass across all sampled locations and years. Both grass percentage nitrogen and grass biomass were log‐transformed as this improved model fit. For samples from 2017 to 2018, each point represents one grazing lawn or off‐lawn location with error bars representing one standard error calculated from the six subsamples collected per replicate. The grazing lawn or off‐lawn location was not recorded for 2016 grass samples analyzed for percentage nitrogen; thus for 2016 samples, we averaged all samples from grazing lawns and off grazing lawns, resulting in only two points from this year. Point shape denotes sampling year, whereas color denotes samples collected on versus off grazing lawns

Grazing lawn soils from 2017 had higher concentrations of phosphorous, sodium, ammonium, and nitrate compared to soils off grazing lawns (Figure 3). Grazing lawns consistently showed decreased total plant biomass, grass biomass, and forb biomass compared to controls over the three years, with levels varying across years (Figure 4b–d and Table S6B–D). Grazing lawn grasses consistently had higher percentage nitrogen (Figure 4a and Table S6A). In contrast, the effect of the 2018 drought was to elevate grass percentage nitrogen uniformly, raising off plot levels to those on grazing lawns in 2016–2017, with grass percentage nitrogen on grazing lawns still ca. 50% higher (Figure 4a and Table S6A). Combined, the decrease in grass percentage nitrogen with increasing grass biomass over lawns and years (Figure 5; marginal *R* ^2^ = 0.19, conditional *R* ^2^ = 0.65, *p* < .001) suggests that increasing biomass diluted grass foliar nitrogen concentrations.

## 4 DISCUSSION

Biotic and abiotic drivers frequently interact to shape community abundance. Here, we show how bison‐grazing lawns in normal precipitation years attract grasshoppers, locally increasing grasshopper abundances. We show this attraction is diminished in a drought year when foliar percentage nitrogen is uniformly higher. Combined, these observations, replicated across 10 bison lawns, suggest nutrient differences as being the driver of grasshopper patch selection. At the same time, five other taxa that collectively represent piercing‐sucking herbivores (herbivorous Hemiptera), omnivores (Formicidae, and Tettigonniidae), and predators (Araneae and parasitoid Hymenoptera) did not accumulate on bison lawns. In an era of human‐altered biogeochemical cycles, variation in nutrient availability may disproportionately affect grasshoppers, a dominant grassland herbivore.

Bison‐grazing lawns altered soil and plant chemistry: soils on grazing lawns had elevated levels of inorganic nitrogen, phosphorus, and sodium and grass foliage had increased nitrogen concentrations relative to nearby locations off grazing lawns. We suggest that acridid grasshoppers chose to forage on these large, high‐quality forage patches for a number of reasons. First, grasshoppers are able to detect and are sensitive to small changes in host plant chemistry (Behmer, 2009; Behmer & Joern, 2008; Jonas & Joern, 2008). Second, grasshopper populations can be limited both by macronutrients such as N, P, and K (Huberty & Denno, 2006; Mattson, 1980) and micronutrients such as Mg and Na (Ibanez et al., 2017; Joern et al., 2012; Welti et al., 2019). Third, individual grasshoppers can have range sizes from ~1 to >200 km^2^ (Otte, 1979), much larger than bison‐grazing lawns, which are generally >50 m^2^ (Catchpole, 1996). Finally, the grazed watershed treatments of KPBS already maintain higher grasshopper abundance (Welti, Qiu, et al., 2019). Combined, all these suggest that the +0.4 to +2.0 *SD* grasshopper abundance on grazing lawns represents local habitat selection rather than higher population abundances.

### 4.1 The complementary effects of drought and grazing lawns on grasshopper abundance

The severe regional drought of 2018, by increasing the background level of foliar nutrients, allowed a second test of the role of nutrient limitation on grasshopper numbers. Grass foliar nitrogen concentrations off grazing lawns during the drought year had similar or greater levels to those on grazing lawns in non‐drought years. This led to (a) overall higher grasshopper abundances in 2018 and (b) a decreased relative attractiveness to the +2% foliar nitrogen levels generated by grazing lawns in a year where grass foliar nitrogen was already elevated off grazing lawns to ca +1.4%. Such positive, but decelerating effect of nutrients on fitness, is a common feature in animal nutrition (Marschner, 2011; McDowell, 2003).

Precipitation can have variable effects grasshopper density and composition (Branson, 2008, 2016; Jonas & Joern, 2007; Jonas et al., 2015). We suggest this is due to the higher densities of foliar macro‐ and micronutrient concentrations in low production, drier prairies (La Pierre & Smith, 2016). As a result, precipitation in drier grasslands produces more plant tissue per unit of precipitation, as plants are less likely to be nutrient‐limited (Belovsky & Slade, 1995; Fielding & Brusven, 1990). In a wetter tallgrass prairie, foliar nutrient concentrations are lower and increases in precipitation are more likely to lower nutrient density to levels limiting to grassland herbivores. Our results for grasshoppers are congruous with the original Plant Stress Hypothesis (White, 1969), stating that drought‐induced increases in foliar nutrient availability benefit herbivorous insects. This is also consistent with a study of 1,910 years of historical records from China, where locust outbreaks were linked to periods of drought (and thus, presumably, higher nutrients, Tian et al., 2011). In other studies finding precipitation decreases grasshopper densities, decreasing plant quality is the primary cited mechanism (Branson, 2008; Capinera & Horton, 1989; Gage & Mukerji, 1977). Finally, one proposed mechanism of the formation and maintenance of grazing lawns is that herbivores induce dry conditions in grazing lawns patches through soil compaction and defoliation (Veldhuis et al., 2014), suggesting plant and grasshopper communities may respond similarly to grazing lawns and drought.

While both plant and herbivore responses to drought vary with ecological community, drought severity, and timing (Harrison et al., 2015; He & Dijkstra, 2014; Huberty & Denno, 2004; Lenhart et al., 2015; Rosenblatt, 2018), precipitation induced plant growth—and with it increased structural carbohydrates— can dilute nitrogen in plant tissue (Loladze, 2014; Welti, Roeder, et al., 2020). Drivers that increase aboveground plant biomass, including increases in precipitation, thus have the potential to create “green deserts” in mixed and tallgrass prairie. Supporting this conclusion, we show a negative relationship between grassland biomass and percent nitrogen in grazing lawn patches over 3 years.

### 4.2 Grasshopper feeding guilds vary in responses to grazing lawns

The forage preference of both the insect and large grazers determines their interaction (Mayengo et al., 2020; van der Plas & Olff, 2014; van Klink et al., 2015). At KPBS bison prefer grasses, reducing the otherwise dominant C_4_ grasses and increasing forb biomass (Collins & Calabrese, 2012) and the abundance and species richness of forb‐feeding grasshoppers (Welti, Qiu, et al., 2019). We found that grasshopper community composition varied with both grazing lawns and between the drought and non‐drought year. Mixed‐feeding (more generalist) grasshoppers accumulated on grazing lawns in both years. Both forbfeeding and grassfeeding grasshoppers showed preference for grazing lawns only in 2019, the non‐drought year, and their shift in abundances drove the grazing lawn‐year interaction. While grass biomass on grazing lawns is low due to high bison consumption, some grassfeeding species—especially *Syrbula admirabilis* (Uhler 1864), *Mermiria picta* (F. Walker, 1870), and *Orphulella speciosa* (Scudder, S.H., 1862)—had a strong preference for the high nutrient grazing lawn patches in the non‐drought year. We hypothesize that the larger number of host plants available to the generalist mixed feeders, allowed them to take advantage of higher nutrient concentrations on grazing lawns in both drought and non‐drought years. Alternatively, as mixed feeders always have more host plant options, they may be less limited by the availability of high nutrient plants, and their attraction to grazing lawns may be a response to plant community composition, rather than nutrient availability. Taxonomic patterns tended to follow feeding guild responses, with the subfamily Gomphocerinae (predominantly grass‐feeders) responding to the interaction between grazing lawns and Melanoplinae (mixed and forb‐feeders) having the strongest response to year, as did forb‐feeders. The Oedipodinae comprise all feeding guilds and were less well sampled; their preference of grazing lawns may also reflect their preference for bareground.

### 4.3 Other taxa did not respond to grazing lawns but increased in the wetter year

In contrast to grasshopper responses, no other collected taxonomic group displayed changes in abundance with respect to grazing lawns. This is particularly notable for the other group of herbivores, the herbivorous Hemiptera. This discrepancy may arise from variation we did not account for, such as different plant species composition on grazing lawns, and/or variable preferences of different Hemiptera taxa which were not identified to lower taxonomic levels. The phloem‐feeding strategy of Hemiptera, in contrast to the tissue‐chewing grasshoppers, may cause differences in which nutrients are available to these taxa, with Hemiptera not having the same access as grasshoppers to the leaf tissue elemental nutrients quantified in this study (Welti et al., 2020). Hemiptera are less mobile than grasshoppers. Additionally, Hemiptera are known to host symbiotic microorganisms, which can provide them with essential amino acids, reducing their need to locate host plants with essential nutrients (Hansen & Moran, 2011). The only non‐grasshopper taxa to vary in abundance with year was the parasitoid Hymenoptera, which had higher abundances in 2019. This response may be due to higher growing season precipitation resulting in more plant aboveground biomass, and thus more habitat volume and our nectar for parasitoids (Post et al., 2000; Welti, Kuczynski, et al., 2020). Generally, non‐grasshopper taxa were only categorized to broad functional‐taxonomic groups and may vary compositionally in response to grazing lawns and drought at finer taxonomic levels. Finally, we note that the presence of large mammalian grazers alters the abundance, diversity, and composition of arthropods, both at KPBS (Welti & Joern, 2018), and in other grasslands (e.g. Farrell et al., 2015; Lind et al., 2017; Zhu et al., 2015). This study does not include non‐grazed grasslands but rather compares grazing lawns within grazed grasslands.

### 4.4 Caveats

There are several limitations of our study, with the most notable being that no plant data are available for 2019. Thus, we are relying on both older plant data from years with comparable rainfall (Figure 4) and previous work to identify the most likely responses of tallgrass prairie foliar nutrient concentrations (Franzke & Reinhold, 2011; Grant et al., 2014; Mayengo et al., 2020; McNaughton, 1984; Prather et al., 2020; Welti, Roeder, et al., 2020). Additionally, arthropod sampling occurred only in July and does not reflect responses from all seasons and species not present during sampling. Grazing lawns and drought can effect grasshoppers in many other ways beyond altering plant nutrients. We cannot rule out that grazing lawns may affect grasshopper abundances through altering soil moisture, microclimate (Prather & Kaspari, 2019), plant diversity (Prather et al., 2020; Welti, Qiu, et al., 2019), and active periods or habitat suitability for natural enemies (Laws & Joern, 2013). Heavy spring precipitation can reduce grasshopper abundances by promoting fungal pathogens (Arthurs et al., 2001). However, these alternative hypotheses do not explain the interaction between grazing lawns and precipitation, which points toward plant nutrient concentrations as the primary driver of grasshopper abundances. As a caveat of this natural experiment, grazing lawn locations were selected by bison and not randomly distributed across the landscape. Thus, while large grazers undoubtedly alter soil properties and plant communities, it is also possible that underlying initial differences led to grazer selection for grazing lawn locations.

### 4.5 Implications and conclusion

Understanding the role of grazing and precipitation on grassland arthropods is key to making a range of policy decisions ranging from biological conservation to agricultural pest management. For example, low intensity or rotational grazing can increase arthropod diversity (van Klink et al., 2015). Diverse grasshopper assemblages, in turn, are less destructive to agricultural crops (Branson et al., 2006). Moreover, climate models (USGCRP, 2018) predict a shift in Great Plains precipitation—from spring to late summer/early fall region—likely reducing plant growth, enhancing plant nutrient density, and enhancing the abundance of KPBS grasshopper populations. These interacting effects of grazing lawns and drought in shaping the nutritional carrying capacity of grasslands in time and space demonstrate the importance of incorporating both abiotic and biotic factors in predicting abundance and species composition.

## CONFLICT OF INTEREST

None declared.

## AUTHOR CONTRIBUTIONS

**Katerina A. Ozment:** Data curation (equal); investigation (equal); methodology (lead); writing–original draft (equal). **Ellen A. R. Welti:** Conceptualization (equal); data curation (equal); formal analysis (equal); investigation (equal); methodology (equal); visualization (equal); writing–original draft (equal). **Monica Shaffer:** Conceptualization (equal); data curation (equal); methodology (equal); writing–review and editing (equal). **Mike Kaspari:** Conceptualization (equal); funding acquisition (equal); investigation (equal); supervision (equal); writing–review and editing (equal).

## Supporting information

Supplementary Material

## Data Availability

Grasshopper and other arthropod taxa abundance, soil chemistry, plant chemistry, and plant biomass data are available in the Dryad Digital Repository: https://doi.org/10.5061/dryad.tqjq2bvz6

## References

[ece37435-bib-0001] Aaltonen, H. , Lindén, A. , Heinonsalo, J. , Biasi, C. , & Pumpanen, J. (2017). Effects of prolonged drought stress on Scots pine seedling carbon allocation. Tree Physiology, 37(4), 418–427. 10.1093/treephys/tpw119 27974653

[ece37435-bib-0002] Arthurs, S. P. , Thomas, M. B. , & Lawton, J. L. (2001). Seasonal patterns of persistence and infectivity of Metarhizium anisopliae var. Acridum in grasshopper cadavers in the Sahel. Entomologia Experimentalis Et Applicata, 100(1), 69–76. 10.1046/j.1570-7458.2001.00849.x

[ece37435-bib-0003] Augustine, D. J. , Blumenthal, D. M. , Springer, T. L. , LeCain, D. R. , Gunter, S. A. , & Derner, J. D. (2018). Elevated CO2 induces substantial and persistent declines in forage quality irrespective of warming in mixedgrass prairie. Ecological Applications: A Publication of the Ecological Society of America, 28(3), 721–735. 10.1002/eap.1680 29297964

[ece37435-bib-0004] Bates, D. , Mächler, M. , Bolker, B. , & Walker, S. (2015). Fitting linear mixed‐effects models using lme4. Journal of Statistical Software, 67(1), 1–48. 10.18637/jss.v067.i01

[ece37435-bib-0005] Behmer, S. T. (2009). Insect herbivore nutrient regulation. Annual Review of Entomology, 54, 165–187. 10.1146/annurev.ento.54.110807.090537 18764740

[ece37435-bib-0006] Behmer, S. T. , & Joern, A. (2008). Coexisting generalist herbivores occupy unique nutritional feeding niches. Proceedings of the National Academy of Sciences, 105(6), 1977–1982. 10.1073/pnas.0711870105 PMC2538867 18238894

[ece37435-bib-0007] Behmer, S. T. , & Joern, A. (2012). Insect herbivore outbreaks viewed through a physiological framework: Insights from Orthoptera. In F. Barbosa , D. Letourneau , & A. Agrawaal (Eds.), Insect outbreaks revisited (pp. 3–29). Academic Press.

[ece37435-bib-0008] Belovsky, G. E. , & Slade, J. B. (1995). Dynamics of two Montana grasshopper populations: Relationships among weather, food abundance and intraspecific competition. Oecologia, 101(3), 383–396. 10.1007/BF00328826 28307061

[ece37435-bib-0009] Branson, D. H. (2008). Influence of a large late summer precipitation event on food limitation and grasshopper population dynamics in a northern Great Plains grassland. Environmental Entomology, 37(3), 686–695.10.1603/0046‐225X(2008)37[686:IOALLS]2.0.CO;2 18559174 10.1603/0046-225x(2008)37[686:ioalls]2.0.co;2

[ece37435-bib-0010] Branson, D. H. (2016). Drought impacts on competition in *Phoetaliotes nebrascensis* (Orthoptera Acrididae) in a northern Mixed Grassland. Environmental Entomology, 45(2), 492–499. 10.1093/ee/nvv225 26744453

[ece37435-bib-0011] Branson, D. H. , Joern, A. , & Sword, G. A. (2006). Sustainable management of insect herbivores in grassland ecosystems: New perspectives in grasshopper control. BioScience, 56(9), 743–755.10.1641/0006‐3568(2006)56[743:SMOIHI]2.0.CO;2

[ece37435-bib-0012] Capinera, J. , & Horton, D. (1989). Geographic variation in effects of weather on grasshopper infestation. Environmental Entomology, 18(1), 8–14. 10.1093/ee/18.1.8

[ece37435-bib-0013] Catchpole, F. B. (1996). The dynamics of bison (Bos bison) grazing patches in tallgrass prairie [Master’s thesis]. Kansas State University.

[ece37435-bib-0014] Collins, S. L. , & Calabrese, L. B. (2012). Effects of fire, grazing and topographic variation on vegetation structure in tallgrass prairie. Journal of Vegetation Science, 23(3), 563–575. 10.1111/j.1654-1103.2011.01369.x

[ece37435-bib-0015] Cornelissen, T. , Wilson Fernandes, G. , & Vasconcellos‐Neto, J. (2008). Size does matter: Variation in herbivory between and within plants and the plant vigor hypothesis. Oikos, 117(8), 1121–1130. 10.1111/j.0030-1299.2008.16588.x

[ece37435-bib-0016] Farrell, K. A. , Harpole, W. S. , Stein, C. , Suding, K. N. , & Borer, E. T. (2015). Grassland arthropods are controlled by direct and indirect interactions with cattle but are largely unaffected by plant provenance. PLoS One, 10(7), e0129823. 10.1371/journal.pone.0129823 26158494 PMC4497643

[ece37435-bib-0017] Fielding, D. J. , & Brusven, M. A. (1990). Historical analysis of grasshopper (Orthoptera: Acrididae) population responses to climate in southern Idaho, 1950–1980. Environmental Entomology, 19(6), 1786–1791. 10.1093/ee/19.6.1786

[ece37435-bib-0018] Franzke, A. , & Reinhold, K. (2011). Stressing food plants by altering water availability affects grasshopper performance. Ecosphere, 2(7), 1–13. 10.1890/ES11-00095.1

[ece37435-bib-0019] Gage, S. H. , & Mukerji, M. K. (1977). A perspective of grasshopper population distribution in Saskatchewan and interrelationship with weather. Environmental Entomology, 6(3), 469–479. 10.1093/ee/6.3.469

[ece37435-bib-0020] Grant, K. , Kreyling, J. , Dienstbach, L. F. H. , Beierkuhnlein, C. , & Jentsch, A. (2014). Water stress due to increased intra‐annual precipitation variability reduced forage yield but raised forage quality of a temperate grassland. Agriculture, Ecosystems & Environment, 186, 11–22. 10.1016/j.agee.2014.01.013

[ece37435-bib-0021] Gutbrodt, B. , Mody, K. , & Dorn, S. (2011). Drought changes plant chemistry and causes contrasting responses in lepidopteran herbivores. Oikos, 120(11), 1732–1740. 10.1111/j.1600-0706.2011.19558.x

[ece37435-bib-0022] Hansen, A. K. , & Moran, N. A. (2011). Aphid genome expression reveals host–symbiont cooperation in the production of amino acids. Proceedings of the National Academy of Sciences, 108(7), 2849–2854. 10.1073/pnas.1013465108 PMC3041126 21282658

[ece37435-bib-0023] Harrison, S. P. , Gornish, E. S. , & Copeland, S. (2015). Climate‐driven diversity loss in a grassland community. Proceedings of the National Academy of Sciences, 112(28), 8672–8677. 10.1073/pnas.1502074112 PMC4507231 26100891

[ece37435-bib-0024] He, M. , & Dijkstra, F. A. (2014). Drought effect on plant nitrogen and phosphorus: A meta‐analysis. The New Phytologist, 204(4), 924–931. 10.1111/nph.12952 25130263

[ece37435-bib-0025] Hempson, G. P. , Archibald, S. , Bond, W. J. , Ellis, R. P. , Grant, C. C. , Kruger, F. J. , Kruger, L. M. , Moxley, C. , Owen‐Smith, N. , Peel, M. J. S. , Smit, I. P. J. , & Vickers, K. J. (2015). Ecology of grazing lawns in Africa. Biological Reviews, 90(3), 979–994. 10.1111/brv.12145 25231416

[ece37435-bib-0026] Huberty, A. F. , & Denno, R. F. (2004). Plant water stress and its consequences for herbivorous insects: A new synthesis. Ecology, 85(5), 1383–1398. 10.1890/03-0352

[ece37435-bib-0027] Huberty, A. F. , & Denno, R. F. (2006). Consequences of nitrogen and phosphorus limitation for the performance of two planthoppers with divergent life‐history strategies. Oecologia, 149(3), 444–455. 10.1007/s00442-006-0462-8 16794833

[ece37435-bib-0028] Ibanez, S. , Millery, A. , D'ottavio, M. , Guilhot, R. , & Vesin, E. (2017). Phosphorus‐rich grasshoppers consume plants high in nitrogen and phosphorus. Ecological Entomology, 42(5), 610–616. 10.1111/een.12425

[ece37435-bib-0029] Joern, A. (2004). Variation in grasshopper (Acrididae) densities in response to fire frequency and bison grazing in tallgrass prairie. Environmental Entomology, 33(6), 1617–1625. 10.1603/0046-225X-33.6.1617

[ece37435-bib-0030] Joern, A. (2005). Disturbance by fire frequency and bison grazing modulate grasshopper assemblages in tallgrass prairie. Ecology, 86(4), 861–873. 10.1890/04-0135

[ece37435-bib-0031] Joern, A. (2018). CGR02 Sweep sampling of grasshoppers on Konza Prairie LTER watersheds. Environmental Data Initiative. 10.6073/pasta/aec67f5d71d14cd39fe8b6b34b4719f4

[ece37435-bib-0032] Joern, A. , & Mole, S. (2005). The plant stress hypothesis and variable responses by blue grama grass (*Bouteloua gracilis*) to water, mineral nitrogen, and insect herbivory. Journal of Chemical Ecology, 31(9), 2069–2090. 10.1007/s10886-005-6078-3 16132213

[ece37435-bib-0033] Joern, A. , Provin, T. , & Behmer, S. T. (2012). Not just the usual suspects: Insect herbivore populations and communities are associated with multiple plant nutrients. Ecology, 93(5), 1002–1015. 10.1890/11-1142.1 22764487

[ece37435-bib-0034] Jonas, J. L. , & Joern, A. (2007). Grasshopper (Orthoptera: Acrididae) communities respond to fire, bison grazing and weather in North American tallgrass prairie: A long‐term study. Oecologia, 153(3), 699–711. 10.1007/s00442-007-0761-8 17546466

[ece37435-bib-0035] Jonas, J. L. , & Joern, A. (2008). Host‐plant quality alters grass/forb consumption by a mixed‐feeding insect herbivore, *Melanoplus bivittatus* (Orthoptera: Acrididae). Ecological Entomology, 33(4), 546–554. 10.1111/j.1365-2311.2008.01004.x

[ece37435-bib-0036] Jonas, J. L. , Wolesensky, W. , & Joern, A. (2015). Weather affects grasshopper population dynamics in continental grassland over annual and decadal periods. Rangeland Ecology & Management, 68(1), 29–39. 10.1016/j.rama.2014.12.011

[ece37435-bib-0037] La Pierre, K. J. , & Smith, M. D. (2016). Soil nutrient additions increase invertebrate herbivore abundances, but not herbivory, across three grassland systems. Oecologia, 180(2), 485–497. 10.1007/s00442-015-3471-7 26474567

[ece37435-bib-0038] Laws, A. N. , & Joern, A. (2013). Predator–prey interactions in a grassland food chain vary with temperature and food quality. Oikos, 122(7), 977–986. 10.1111/j.1600-0706.2012.20419.x

[ece37435-bib-0039] Legendre, P. , & Anderson, M. J. (1999). Distance‐based redundancy analysis: Testing multispecies responses in multifactorial ecological experiments. Ecological Monographs, 69(1), 1–24.10.1890/0012‐9615(1999)069[0001:DBRATM]2.0.CO;2

[ece37435-bib-0040] Lenhart, P. A. , Eubanks, M. D. , & Behmer, S. T. (2015). Water stress in grasslands: Dynamic responses of plants and insect herbivores. Oikos, 124(3), 381–390. 10.1111/oik.01370

[ece37435-bib-0041] Lind, E. M. , La Pierre, K. J. , Seabloom, E. W. , Alberti, J. , Iribarne, O. , Firn, J. , Gruner, D. S. , Kay, A. D. , Pascal, J. , Wright, J. P. , Yang, L. , & Borer, E. T. (2017). Increased grassland arthropod production with mammalian herbivory and eutrophication: A test of mediation pathways. Ecology, 98(12), 3022–3033. 10.1002/ecy.2029 28940315

[ece37435-bib-0042] Loladze, I. (2014). Hidden shift of the ionome of plants exposed to elevated CO2 depletes minerals at the base of human nutrition. Elife, 3, e02245. 10.7554/eLife.02245 24867639 PMC4034684

[ece37435-bib-0043] Luo, W. , Xu, C. , Ma, W. , Yue, X. , Liang, X. , Zuo, X. , Knapp, A. K. , Smith, M. D. , Sardans, J. , Dijkstra, F. A. , Peñuelas, J. , Bai, Y. , Wang, Z. , Yu, Q. , & Han, X. (2018). Effects of extreme drought on plant nutrient uptake and resorption in rhizomatous vs bunchgrass‐dominated grasslands. Oecologia, 188(2), 633–643. 10.1007/s00442-018-4232-1 30043231

[ece37435-bib-0044] Marschner, H. (2011). Mineral nutrition of higher plants (3rd ed.). Elsevier. Retrieved from https://www.elsevier.com/books/marschners‐mineral‐nutrition‐of‐higher‐plants/marschner/978‐0‐12‐384905‐2

[ece37435-bib-0045] Mattson, W. J. (1980). Herbivory in relation to plant nitrogen content. Annual Review of Ecology and Systematics, 11(1), 119–161. 10.1146/annurev.es.11.110180.001003

[ece37435-bib-0046] Mayengo, G. , Piel, A. , & Treydte, A. C. (2020). The importance of nutrient hotspots for grazing ungulates in a Miombo ecosystem, Tanzania. PLoS One, 15, e0230192. 10.1371/journal.pone.0230192 32226036 PMC7105114

[ece37435-bib-0047] McDowell, L. R. (Ed.). (2003). Minerals in animal and human nutrition (2nd ed.). Elsevier.

[ece37435-bib-0048] McNaughton, S. J. (1984). Grazing lawns: Animals in herds, plant form, and coevolution. The American Naturalist, 124(6), 863–886.JSTOR.

[ece37435-bib-0049] Oksanen, J. , Blanchet, F. G. , Friendly, M. , Kindt, R. , Legendre, P. , McGlinn, D. , Minchin, P. R. , O'hara, R. B. , Simpson, G. L. , & Solymos, P. (2018). Vegan: Community Ecology Package, R package, v. 2.4–6.

[ece37435-bib-0050] Onsager, J. A. (2000). Suppression of grasshoppers in the Great Plains through grazing management. Rangeland Ecology & Management, 53(6), 592–602. 10.2307/4003152

[ece37435-bib-0051] Otte, D. (1979). Biogeographic patterns in flight capacity of Nearctic grasshoppers. Entomological News, 90(4), 153–158.

[ece37435-bib-0052] Post, D. M. , Pace, M. L. , & Hairston, N. G. (2000). Ecosystem size determines food‐chain length in lakes. Nature, 405(6790), 1047–1049. 10.1038/35016565 10890443

[ece37435-bib-0053] Prather, R. M. , Castillioni, K. , Welti, E. A. R. , Kaspari, M. , & Souza, L. (2020). Abiotic factors and plant biomass, not plant diversity, strongly shape grassland arthropods under drought conditions. Ecology, 101(6), e03033. 10.1002/ecy.3033 32112407

[ece37435-bib-0054] Prather, R. M. , & Kaspari, M. (2019). Plants regulate grassland arthropod communities through biomass, quality, and habitat heterogeneity. Ecosphere, 10(10), e02909. 10.1002/ecs2.2909

[ece37435-bib-0055] Purdon, J. , Parr, C. L. , & Somers, M. J. (2019). Grazing by large savanna herbivores indirectly alters ant diversity and promotes resource monopolisation. PeerJ, 7, e6226. 10.7717/peerj.6226 30648021 PMC6330944

[ece37435-bib-0056] R Core Team (2020). R: A language and environment for statistical computing (4.0.3) [Computer software]. R Foundation for Statistical Computing. Retrieved from http://www.R‐project.org/

[ece37435-bib-0057] Raynor, E. J. , Joern, A. , Nippert, J. B. , & Briggs, J. M. (2016). Foraging decisions underlying restricted space use: Effects of fire and forage maturation on large herbivore nutrient uptake. Ecology and Evolution, 6(16), 5843–5853. 10.1002/ece3.2304 27547359 PMC4983596

[ece37435-bib-0058] Rosenblatt, A. E. (2018). Shifts in plant nutrient content in combined warming and drought scenarios may alter reproductive fitness across trophic levels. Oikos, 127(12), 1853–1862. 10.1111/oik.05272

[ece37435-bib-0059] Shaffer, M. (2019). Ecology of grazing lawns on tallgrass prairie [Division of Biology, Kansas State University]. Retrieved from https://krex.k‐state.edu/dspace/handle/2097/39462

[ece37435-bib-0060] Steinauer, E. M. , & Collins, S. L. (1995). Effects of urine deposition on small‐scale patch structure in prairie vegetation. Ecology, 76(4), 1195–1205. 10.2307/1940926

[ece37435-bib-0061] Tian, H. , Stige, L. C. , Cazelles, B. , Kausrud, K. L. , Svarverud, R. , Stenseth, N. C. , & Zhang, Z. (2011). Reconstruction of a 1,910‐y‐long locust series reveals consistent associations with climate fluctuations in China. Proceedings of the National Academy of Sciences, 108(35), 14521–14526. 10.1073/pnas.1100189108 PMC3167559 21876131

[ece37435-bib-0062] USGCRP (2018). Impacts, risks, and adaptation in the United States: Fourth National Climate Assessment (Vol. II). U.S. Global Change Research Program. Retrieved from https://nca2018.globalchange.gov

[ece37435-bib-0063] van der Plas, F. , & Olff, H. (2014). Mesoherbivores affect grasshopper communities in a megaherbivore‐dominated South African savannah. Oecologia, 175(2), 639–649. 10.1007/s00442-014-2920-z 24705648

[ece37435-bib-0064] van Klink, R. , Rickert, C. , Vermeulen, R. , Vorst, O. , WallisDeVries, M. F. , & Bakker, J. P. (2013). Grazed vegetation mosaics do not maximize arthropod diversity: Evidence from salt marshes. Biological Conservation, 164, 150–157. 10.1016/j.biocon.2013.04.023

[ece37435-bib-0065] van Klink, R. , van der Plas, F. , van Noordwijk, C. G. E. (. T. , WallisDeVries, M. F. , & Olff, H. (2015). Effects of large herbivores on grassland arthropod diversity. Biological Reviews, 90(2), 347–366. 10.1111/brv.12113 24837856 PMC4402009

[ece37435-bib-0066] Veldhuis, M. P. , Fakkert, H. F. , Berg, M. P. , & Olff, H. (2016). Grassland structural heterogeneity in a savanna is driven more by productivity differences than by consumption differences between lawn and bunch grasses. Oecologia, 182(3), 841–853. 10.1007/s00442-016-3698-y 27522607 PMC5042998

[ece37435-bib-0067] Veldhuis, M. P. , Howison, R. A. , Fokkema, R. W. , Tielens, E. , & Olff, H. (2014). A novel mechanism for grazing lawn formation: Large herbivore‐induced modification of the plant–soil water balance. Journal of Ecology, 102(6), 1506–1517. 10.1111/1365-2745.12322

[ece37435-bib-0068] Welti, E. A. R. , & Joern, A. (2018). Fire and grazing modulate the structure and resistance of plant–floral visitor networks in a tallgrass prairie. Oecologia, 186(2), 517–528. 10.1007/s00442-017-4019-9 29197973

[ece37435-bib-0069] Welti, E. A. R. , Kuczynski, L. , Marske, K. , Sanders, N. J. , de Beurs, K. M. , & Kaspari, M. (2020). Salty, mild, and low plant biomass grasslands increase top‐heaviness of invertebrate trophic pyramids. Global Ecology and Biogeography, 29(9), 1474–1485. 10.1111/GEB.13119

[ece37435-bib-0070] Welti, E. A. R. , Prather, R. M. , Sanders, N. J. , de Beurs, K. M. , & Kaspari, M. (2020). Bottom‐up when it is not top‐down: Predators and plants control biomass of grassland arthropods. Journal of Animal Ecology, 89(5), 1286–1294. 10.1111/1365-2656.13191 32115723

[ece37435-bib-0071] Welti, E. A. R. , Qiu, F. , Tetreault, H. M. , Ungerer, M. , Blair, J. , & Joern, A. (2019). Fire, grazing and climate shape plant‐grasshopper interactions in a tallgrass prairie. Functional Ecology, 33(4), 735–745. 10.1111/1365-2435.13272

[ece37435-bib-0072] Welti, E. A. R. , Roeder, K. A. , de Beurs, K. M. , Joern, A. , & Kaspari, M. (2020). Nutrient dilution and climate cycles underlie declines in a dominant insect herbivore. Proceedings of the National Academy of Sciences of the United States of America, 117(13), 7271–7275. 10.1073/pnas.1920012117 32152101 PMC7132292

[ece37435-bib-0073] Welti, E. A. R. , Sanders, N. J. , de Beurs, K. M. , & Kaspari, M. (2019). A distributed experiment demonstrates widespread sodium limitation in grassland food webs. Ecology, 100(3), e02600. 10.1002/ecy.2600 30726560

[ece37435-bib-0074] White, T. C. R. (1969). An index to measure weather‐induced stress of trees associated with outbreaks of Psyllids in Australia. Ecology, 50(5), 905–909.JSTOR. 10.2307/1933707

[ece37435-bib-0075] Zhu, L. , Johnson, D. , Wang, W. , Ma, L. , & Rong, Y. (2015). Grazing effects on carbon fluxes in a Northern China grassland. Journal of Arid Environments, 114, 10.1016/j.jaridenv.2014.11.004

[ece37435-bib-0076] Zuur, A. , Ieno, E. N. , Walker, N. , Saveliev, A. A. , & Smith, G. M. (2009). Mixed effects models and extensions in ecology with R. Springer‐Verlag. 10.1007/978-0-387-87458-6

