# Supplementary material for: Tracking nutrients in space and time: Interactions between grazing lawns and drought drive abundances of tallgrass prairie grasshoppers
Source: Ecol Evol. 2021 Mar 18;11(10):5413–23. doi: 10.1002/ece3.7435 (PMC8131794; doi:10.1002/ece3.7435)
Supplement: Supplementary file 1 — Supplementary Material [file ECE3-11-5413-s001.docx]

**Supporting Information**

Tracking nutrients in space and time: Interactions between grazing lawns and drought drive abundances of tallgrass prairie grasshoppers

Katerina A. Ozment, Ellen A. R. Welti, Monica Shaffer, Michael Kaspari

Ecology & Evolution 2021

**Contents:**

Table S1. Grazing lawn information.

Table S2. Grasshopper data summary.

Table S3. Mixed models of grasshopper feeding guilds and families.

Table S4. Non-acridid taxa mixed models.

Table S5. Redundancy Analysis ANOVA.

Table S6. Plant models.

Fig. S1. Temperature and precipitation.

Fig. S2. Photo of a grazing lawn.

Fig. S3. Grasshopper feeding guilds and family results.

**Supplemental Tables**

**Table S1.** Grazing lawn pair identification number, watershed, if the lawn pair was included in arthropod sampling, geographic location, burn treatment frequency, and last year the watershed was burned prior to sampling for the 13 paired on and off grazing lawn sites included in our study.

| Grazing lawn pair ID | Watershed | Sampled for arthropods | Latitude | Longitude | Fire return interval | Last year burned |
| --- | --- | --- | --- | --- | --- | --- |
| 1 | N4A | yes | 39.077383 | -96.60153 | 4 | 2016 |
| 2 | N4A | no | 39.07742 | -96.60263 | 4 | 2016 |
| 3 | N4D | no | 39.077388 | -96.58655 | 4 | 2017 |
| 4 | N4D | no | 39.073723 | -96.58270 | 4 | 2017 |
| 5 | N4D | yes | 39.085581 | -96.58479 | 4 | 2017 |
| 6 | N4A | yes | 39.083859 | -96.60846 | 4 | 2016 |
| 7 | N2B | yes | 39.086301 | -96.58731 | 2 | 2017, 2019 |
| 8 | N4C | yes | 39.091417 | -96.60433 | 4 | 2018 |
| 9 | N2A | yes | 39.098645 | -96.60479 | 2 | 2018 |
| 10 | N4A | yes | 39.079941 | -96.59974 | 4 | 2016 |
| 11 | N20A | yes | 39.089539 | -96.59626 | 20 | 2011 |
| 12 | N4B | yes | 39.095533 | -96.57828 | 4 | 2015, 2019 |
| 13 | N4A | yes | 39.083392 | -96.60282 | 4 | 2016 |

**Table S2.** Grasshopper abundances by species collected on and off grazing lawns in 2018 and 2019. Unlisted are grasshoppers which were not identified to species (primarily very small nymphs) are 2 *Hesperotettix* spp, 7 *Melanoplus* spp, and 13 Oedipodinae spp. The species are ordered by subfamily (Gomphocerinae, Melanoplinae, and Oedipodinae), and then in alphabetical order.

| **Grasshopper species** | **Off lawn 2018** | **On lawn 2018** | **Off lawn 2019** | **On lawn 2019** |
| --- | --- | --- | --- | --- |
| *Ageneotettix deorum* | 0 | 6 | 0 | 1 |
| *Boopedon gracile* | 1 | 1 | 0 | 3 |
| *Mermiria bivittata* | 1 | 0 | 3 | 1 |
| *Mermiria picta* | 4 | 3 | 3 | 12 |
| *Orphulella speciosa* | 52 | 75 | 8 | 68 |
| *Syrbula admirabilis* | 13 | 18 | 1 | 93 |
| *Campylacantha olivacea* | 46 | 41 | 5 | 22 |
| *Hesperotettix speciosus* | 13 | 3 | 11 | 9 |
| *Hesperotettix viridis* | 4 | 2 | 18 | 3 |
| *Hypochlora alba* | 15 | 5 | 8 | 21 |
| *Melanoplus bivittatus* | 4 | 1 | 9 | 6 |
| *Melanoplus femurrubrum* | 61 | 168 | 13 | 109 |
| *Melanoplus keeleri* | 73 | 50 | 30 | 69 |
| *Melanoplus packardii* | 0 | 2 | 0 | 1 |
| *Melanoplus scudderi* | 29 | 25 | 15 | 10 |
| *Phoetaliotes nebrascensis* | 34 | 28 | 10 | 22 |
| *Arphia xanthoptera* | 1 | 8 | 2 | 2 |
| *Pardalophora haldemanii* | 0 | 0 | 0 | 3 |
| *Hadrotettix trifasciatus* | 0 | 2 | 0 | 0 |
| *Chortophaga viridifasciata* | 0 | 0 | 0 | 1 |
| *Xanthippus corallipes* | 0 | 0 | 1 | 0 |

**Table S3.** Mixed model results for effects of grazing lawn (lawn) and year on abundances of the grasshopper feeding guilds of mixed-feeders (A), grass-feeders (B), and forb-feeders (C), and abundances within subfamilies including Gomphocerinae (D), Melanoplinae (E), and Oedipodinae (F), with n=40 for all models. Numbers in parentheses indicate total number of individuals sampled. Table includes estimate, estimate standard error, t-test statistic, and p-value for each fixed variable and the intercept. Positive estimates for lawn indicate higher abundances on versus off grazing lawns. Positive estimates for year indicate higher abundances in 2018 (the drought year) compared to 2019. Abundance responses are visualized in Fig. S3.

|  | **Estimate** | **Std. Error** | **t** | ***P*** |
| --- | --- | --- | --- | --- |
| **A.) Mixed-feeding grasshoppers (374)** | | | | |
| Intercept | 0.66 | 0.15 | 4.32 | <0.001 |
| lawn | 0.33 | 0.15 | 2.25 | 0.02 |
| year | 0.26 | 0.15 | 1.79 | 0.07 |
| lawn*year | 0.02 | 0.21 | 0.1 | 0.92 |
| **B.) Grass-feeding grasshoppers (465)** | | | | |
| Intercept | 0.99 | 0.09 | 10.3 | <0.001 |
| lawn | 0.02 | 0.11 | 0.15 | 0.88 |
| year | 0.53 | 0.11 | 4.88 | <0.001 |
| lawn*year | 0.8 | 0.15 | 5.23 | <0.001 |
| **C.) Forb-feeding grasshoppers (565)** | | | | |
| Intercept | 1.26 | 0.09 | 14.15 | <0.001 |
| lawn | -0.13 | 0.13 | -1.01 | 0.31 |
| year | 0.37 | 0.13 | 2.91 | 0.004 |
| lawn*year | 0.35 | 0.18 | 1.97 | 0.049 |
| **D.) Gomphocerinae (367)** | | | | |
| Intercept | 0.78 | 0.11 | 7.36 | <0.001 |
| lawn | 0.15 | 0.13 | 1.19 | 0.23 |
| year | 0.46 | 0.13 | 3.67 | <0.001 |
| lawn*year | 0.74 | 0.18 | 4.16 | <0.001 |
| **E.) Melanoplinae (1004)** | | | | |
| Intercept | 1.42 | 0.07 | 19.02 | <0.001 |
| lawn | 0.04 | 0.1 | 0.39 | 0.7 |
| year | 0.36 | 0.1 | 3.54 | <0.001 |
| lawn*year | 0.26 | 0.14 | 1.83 | 0.07 |
| **F.) Oedipodinae (33)** | | | | |
| Intercept | 0.06 | 0.06 | 0.94 | 0.35 |
| lawn | 0.27 | 0.09 | 3 | 0.003 |
| year | -0.02 | 0.09 | -0.2 | 0.85 |
| lawn*year | -0.04 | 0.13 | -0.33 | 0.74 |

**Table S4.** Mixed model results for effects of grazing lawn (lawn) and year on abundances of herbivorous Hemiptera (A), Tettigoniidae (B), Formicidae (C), parasitoid Hymenoptera (D), and Araneae (E), with n=40 for all models. Numbers in parentheses indicate total number of individuals sampled. Table includes estimate, estimate standard error, t-test statistic, and p-value for each fixed variable and the intercept. Positive estimates for lawn indicate higher abundances on versus off grazing lawns. Positive estimates for year indicate higher abundances in 2018 (the drought year) compared to 2019.

|  | **Estimate** | **Std. Error** | **t** | ***P*** |
| --- | --- | --- | --- | --- |
| **A.) Herbivorous Hemiptera (2169)** | | | | |
| Intercept | 1.54 | 0.11 | 14.33 | <0.001 |
| lawn | 0.05 | 0.15 | 0.35 | 0.72 |
| year | 0.01 | 0.15 | 0.08 | 0.94 |
| lawn*year | 0.22 | 0.21 | 1.01 | 0.31 |
| **B.) Tettigoniidae (166)** | | | | |
| Intercept | 0.61 | 0.1 | 6.16 | <0.001 |
| lawn | 0.03 | 0.14 | 0.24 | 0.81 |
| year | 0.13 | 0.14 | 0.96 | 0.34 |
| lawn*year | 0.21 | 0.2 | 1.06 | 0.29 |
| **C.) Formicidae (319)** | | | | |
| Intercept | 0.48 | 0.15 | 3.24 | 0.001 |
| lawn | 0.3 | 0.2 | 1.52 | 0.13 |
| year | -0.24 | 0.2 | -1.24 | 0.21 |
| lawn*year | -0.18 | 0.28 | -0.64 | 0.52 |
| **D.) Parasitoid Hymenoptera (320)** | | | | |
| Intercept | 0.12 | 0.09 | 1.28 | 0.2 |
| lawn | 0.04 | 0.13 | 0.27 | 0.79 |
| year | -1 | 0.13 | -7.6 | <0.001 |
| lawn*year | -0.14 | 0.19 | -0.72 | 0.47 |
| **E.) Araneae (837)** | | | | |
| Intercept | 1.29 | 0.09 | 13.79 | <0.001 |
| lawn | -0.21 | 0.13 | -1.59 | 0.11 |
| year | -0.11 | 0.13 | -0.8 | 0.42 |
| lawn*year | 0.12 | 0.19 | 0.65 | 0.52 |

**Table S5.** ANOVA test statistics for the partial RDA of grasshopper species composition shown in Fig. 2. Fixed effects include if the sample was on or off a grazing lawn (lawn) and year the sample was collected. The RDA is conditional on the grazing lawn pair identity. F distributions and corresponding p-values were generated using 999 permutations.

|  | **df** | **Variance** | **F** | ***P*** |
| --- | --- | --- | --- | --- |
| lawn | 1 | 1.47 | 3.04 | 0.001 |
| year | 1 | 1.18 | 2.44 | 0.002 |
| Residual | 28 | 13.52 |  |  |

**Table S6.** Linear mixed model results including estimate, standard error (SE), t-value, and p-value from grass % nitrogen (A), total plant biomass (B), grass biomass (C), and forb biomass (D) comparisons across grazing lawns versus off lawn samples and year of sample. A visualization of these results is provided as boxplots in Fig. 4.

| **comparison** | **Estimate** | **SE** | **t-value** | ***P*** |
| --- | --- | --- | --- | --- |
| **A.) Grass %N** |  |  |  |  |
| on v off lawn | 0.31 | 0.03 | 10.24 | <0.001 |
| 2016 v 2017 | 0.04 | 0.11 | 0.38 | 0.7 |
| 2016 v 2018 | -0.41 | 0.11 | 3.82 | <0.001 |
| 2017 v 2018 | -0.45 | 0.05 | -8.38 | <0.001 |
| **B.) Total plant biomass** | | | | |
| on v off lawn | -121.5 | 10.5 | -11.6 | <0.001 |
| 2016 v 2017 | 56 | 15 | 3.7 | <0.001 |
| 2016 v 2018 | 114.9 | 15 | 7.6 | <0.001 |
| 2017 v 2018 | 58.9 | 14.4 | 4.1 | <0.001 |
| **C.) Grass biomass** | | | | |
| on v off lawn | -68.9 | 9.3 | -7.4 | <0.001 |
| 2016 v 2017 | 109.2 | 15.2 | 7.2 | <0.001 |
| 2016 v 2018 | 142.3 | 15.2 | 9.4 | <0.001 |
| 2017 v 2018 | 33.1 | 14.8 | 2.2 | 0.03 |
| **D.) Forb biomass** | | | | |
| on v off lawn | -52.6 | 6.1 | -8.6 | <0.001 |
| 2016 v 2017 | 52.6 | 10.4 | 5.1 | <0.001 |
| 2016 v 2018 | 24.3 | 10.4 | 2.3 | 0.02 |
| 2017 v 2018 | 28.3 | 10.1 | 2.8 | 0.005 |

**Supplemental Figures**

**
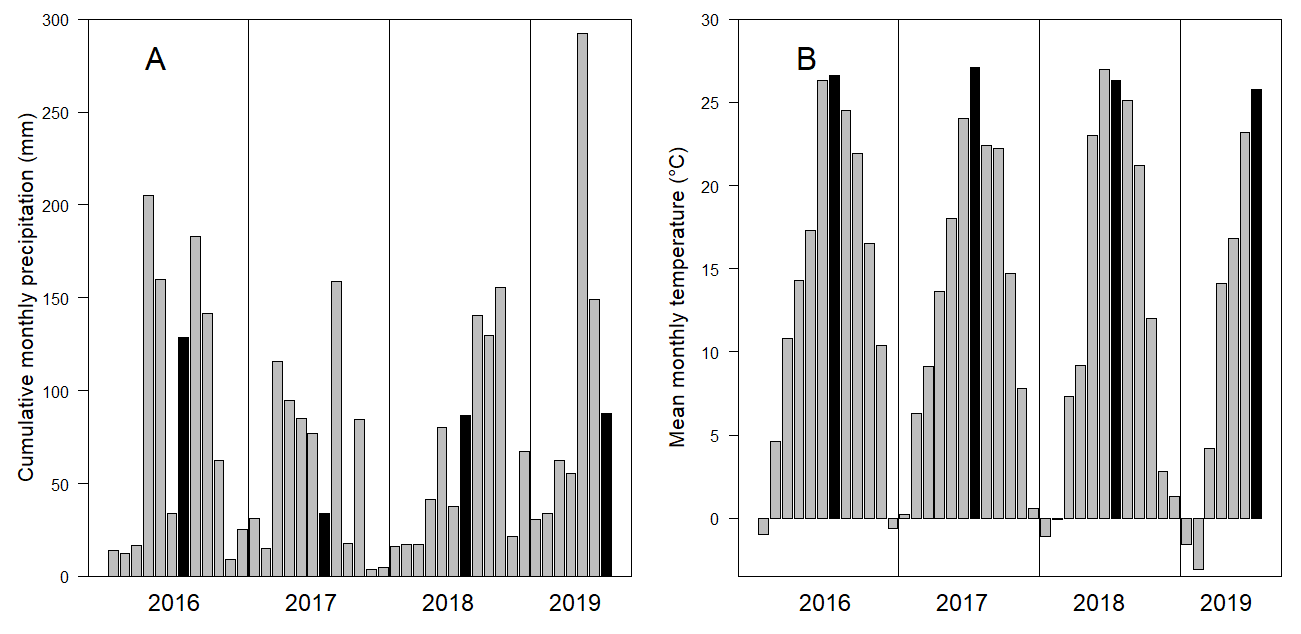
**

**Figure S1.** Monthly cumulative precipitation (A) and monthly mean temperature (B) from January 2017 to the last month of arthropod sampling, July 2019. Black bars indicate July, the month in which arthropod sampling occurred in 2018 and 2019.


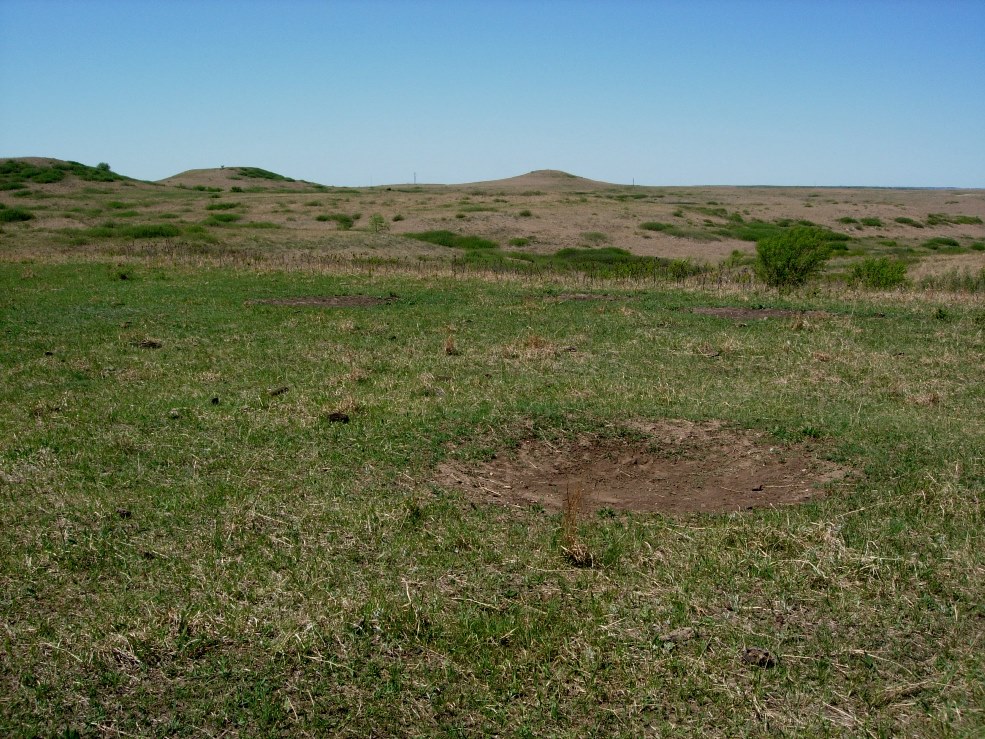


**Figure S2.** A bison grazing lawn on Konza Prairie Biological Station (KPBS) in spring (photo credit: Eva A. Horne). The short vegetation stature and intense greenness of grazing lawns in spring makes them easy to spot.


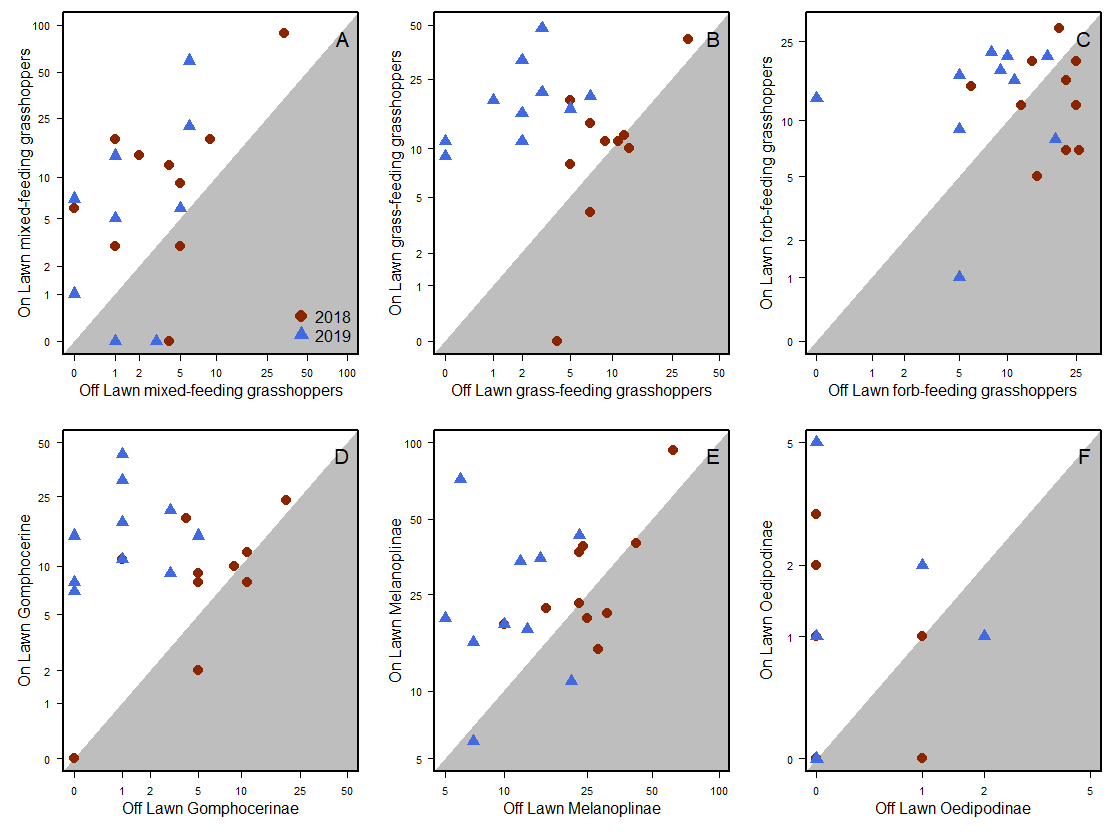


**Figure S3.** Paired comparison of sampled abundance on and off grazing lawns separated by feeding guilds of mixed-feeding (A), grass-feeding (B), and forb-feeding (C) grasshoppers and by the subfamilies Gomphocerinae (D), Melanoplinae (E), and Oedipodinae (F). Model statistics for abundance responses to grazing lawn and year are provided in Table S3.
